# Application of Bioactive Hydrogels for Functional Treatment of Intrauterine Adhesion

**DOI:** 10.3389/fbioe.2021.760943

**Published:** 2021-09-21

**Authors:** Jingying Wang, Chao Yang, Yuxin Xie, Xiaoxu Chen, Ting Jiang, Jing Tian, Sihui Hu, Yingli Lu

**Affiliations:** Department of Obstetrics and Gynecology, The Second Hospital, Jilin University, Changchun, China

**Keywords:** bioactive hydrogel, reproductive medicine, drug delivery system, stem cell therapy, intrauterine adhesion

## Abstract

Intrauterine adhesion (IUA) is a common endometrial disease and one of the main causes of infertility in women of childbearing age. Current treatment strategies, such as hysteroscopic adhesion resection, hysteroscopic transcervical resection of adhesion (TCRA), the use of local hormone drugs, and anti-adhesion scaffold implantation, do not provide a satisfactory pregnancy outcome for moderate-severe IUA, which presents a great challenge in reproductive medicine. With the development of material engineering, various bioactive and functional hydrogels have been developed using natural and synthetic biomaterials. These hydrogels are not only used as barely physical barriers but are also designed as vectors of hormone drugs, growth factors, and stem cells. These characteristics give bioactive hydrogels potentially important roles in the prevention and treatment of IUA. However, there is still no systematic review or consensus on the current advances and future research direction in this field. Herein, we review recent advances in bioactive hydrogels as physical anti-adhesion barriers, *in situ* drug delivery systems, and 3D cell delivery and culture systems for seeded cells in IUA treatment. In addition, current limitations and future perspectives are presented for further research guidance, which may provide a comprehensive understanding of the application of bioactive hydrogels in intrauterine adhesion treatment.

## Introduction

The uterus is composed of three tissue layers, namely, the endometrium, myometrium, and perimetrium (Mancini and Pensabene, 2019). The endometrium is the innermost layer composed of epithelial and stromal components; it is a unique tissue that undergoes a repetitive cycle of cell proliferation, differentiation, and shedding during the reproductive years of a woman’s life, providing the “fertile ground” for embryo implantation (Lv et al., 2021). Intrauterine adhesion (IUA) is a result of endometrial injury and infection caused by unsafe abortion and poor maternal care, which can lead to partial or complete occlusion of the uterine cavity, resulting in periodic abdominal pain, oligomenorrhea, amenorrhea, even infertility (Liao et al., 2021). A normal endometrium is a determinant of fertility, and IUA is recorded to be the second most common cause of female infertility after fallopian tube obstruction (Wei et al., 2020). As for IUA treatment, current therapies include hysteroscopic adhesiolysis, TCRA, hormonal therapy, and anti-adhesion material implantation, including the intrauterine device (IUD) (Huang et al., 2020a; Han et al., 2020). Although current treatment strategies have achieved positive effects in some cases, the prognosis of patients with severe IUA is unsatisfactory, and the incidence of re-adhesion after the operation is still high (Young and Evans-Hoeker, 2014). Thus, patients always accept adjuvant therapy after operation to avoid the recurrence of re-adhesion after surgery (Li et al., 2021). To date, clinicians have already applied biomaterials such as hyaluronic acid (HA) and INTERCEED^®^—an absorbable adhesion barrier made of oxidized regenerated cellulose—after surgery in IUA patients to prevent postoperative re-adhesion (Zhang et al., 2020a). In addition, numerous adjuvant therapies and biomaterials, including bioactive hydrogels, can be selected for those patients who suffer from severe IUA. With the rapid development of regenerative medicine and tissue engineering, in addition to applying traditional adjuvant therapy to IUA patients, clinicians are also seeking the potential of endometrial regeneration through tissue engineering.

Hydrogels are formed by water-soluble or hydrophilic polymers through certain chemical or physical cross-linking, and are composed of a hydrophilic three-dimensional (3D) network structure. The hydrogels swell rapidly in water and can retain a large volume of water without dissolving in this swollen state, which is very similar to soft tissue (Milcovich et al., 2017; Manna et al., 2019). Hydrogels are widely applied in the field of tissue engineering owing to their excellent properties in drug release, 3D cell culture, and simulation of an extracellular matrix (Chang et al., 2018). In the treatment of IUA, the bioactive hydrogels show potential in anti-adhesion and endometrial reconstruction. In order to improve the biocompatibility, reduce the potential cytotoxicity, and adapt to the special pathological microenvironment of the IUA, researchers have further constructed and optimized hydrogels (Tang et al., 2018). Although hydrogels have performed outstandingly in tissue regeneration, cell culture, and drug delivery, their application in the field of reproductive medicine is still in the exploratory stage. Only a limited number of hydrogel products and technologies have been successfully used in humans (Huang and Ding, 2019).

**SCHEME 1:**
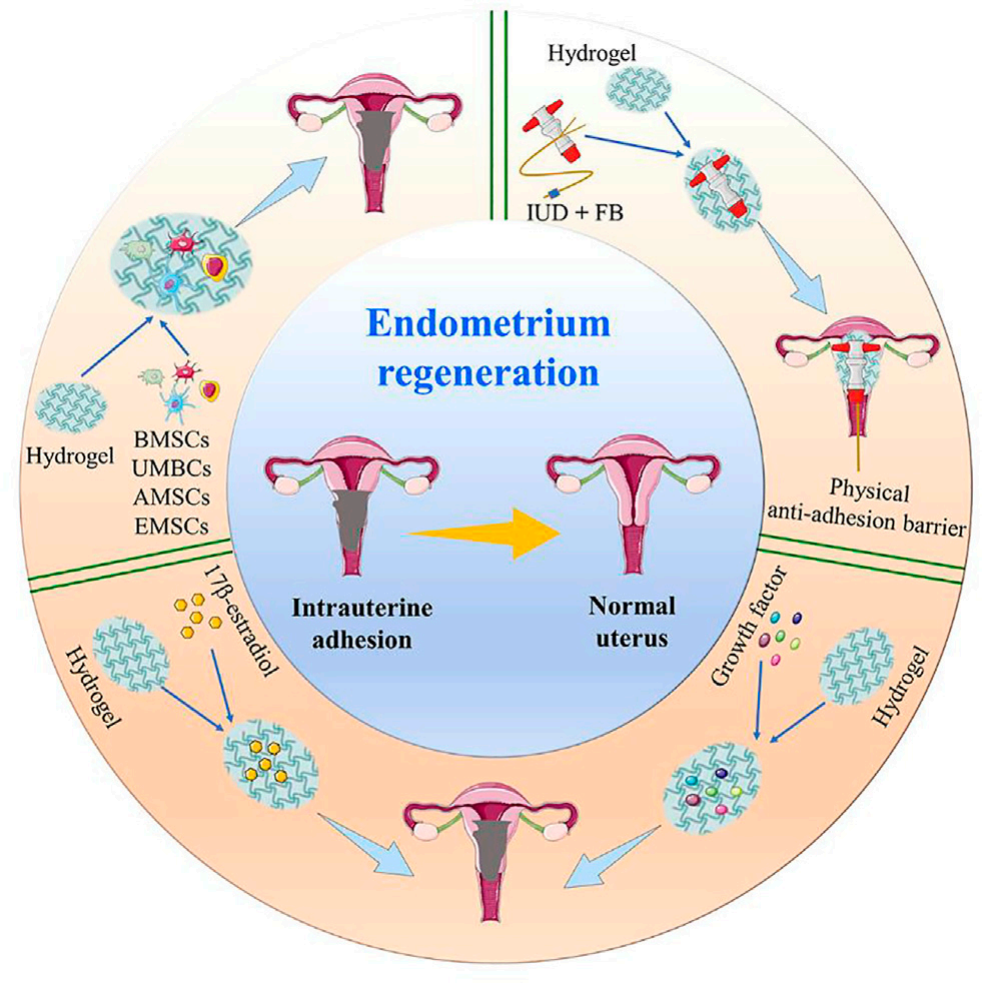
Advances in bioactive hydrogels as a therapeutic option for IUA.

In this review, we summarize the latest advances in hydrogels as a therapeutic option for IUA. This article focuses on the latest research regarding hydrogels as physical anti-adhesion barriers, *in situ* drug delivery systems, and 3D cell delivery and culture systems, in order to improve their application in IUA (Scheme 1).

## The Design of Hydrogels for IUA

The design of hydrogels for biomedical applications needs to consider the purpose of the application and the disease-specific microenvironment. IUA can be characterized as endometrial fibrosis, where the intrauterine walls partially or completely adhere to each other, thus resulting in narrowing or disappearance of the uterine cavity (Carbonnel et al., 2021). The characteristic pathological change of IUA is endometrial injury, which has become a primary factor resulting in oligomenorrhea and related complications (Xu et al., 2020). During natural repair of an endometrial lesion, the luminal epithelial cells are largely lost and are replaced by inactive columnar endometrial epithelium, the stroma is replaced by fibrous tissue, the endometrium becomes significantly thinner and loses response to estrogen and progesterone (Zhao et al., 2016). As mentioned previously, the major aim of the application of bioactive hydrogels is to prevent the recurrence of adhesion and to promote endometrial regeneration. To be an ideal biomaterial for patients with IUA, biocompatibility, immunogenicity, mechanics, degradability, the possibility of infection and transmission of disease, the ability to promote stem cell recruitment, intimal repair and reproductive function recovery, drug-controlled release, availability and even ethical issues should be taken into consideration (López-Martínez et al., 2021). Therefore, the design strategy for bioactive hydrogels should consider the purpose of the application and the disease-specific microenvironment of IUA.

Firstly, one of the main characteristics of the hydrogels used for anti-IUA barrier materials is its ability to form a mechanical intrauterine cavity to decrease the formation of fibrous tissue. The uterine cavity is surrounded by a muscular layer of interlacing smooth muscle fibers, which usually causes the uterine cavity to collapse during the postoperative healing process unless it is mechanically distended. Since the uterine cavity is varied in size and shape, the bioactive hydrogels which can adjust themselves to different uterine cavities may fully promote endometrium regeneration. Moreover, the speed of degradation of the hydrogels needs to be manageable to avoid rapid degradation because they must remain in the uterine cavity for a certain time to resist adhesion recurrence (Wang et al., 2020a). After the healing process is completed, the bioactive hydrogels as barriers must be degraded or absorbed naturally, rather than remaining as a foreign body. Among the hydrogel systems sensitive to stimuli, thermosensitive hydrogels are the most extensively studied. Depending on the properties, thermosensitive matrices are divided into systems showing lower critical solution temperature (LCST) or upper critical solution temperature (UCST) (Kasiński et al., 2020). In this field, temperature-responsive hydrogels have attracted the attention of researchers; especially the hydrogels that are liquid at room temperature and can be rapidly gelled at physiological temperature around specific tissues (Kapoor and Kundu, 2016). The most important hydrogels for biomedical applications are systems with LCST close to the physiological temperature. This type of hydrogel is widely used in anti-adhesion materials because of the ease of its control and its rapid response to physical and chemical changes (Keskin et al., 2019). Compared to other hydrogels, temperature-responsive hydrogels can mechanically help shape the normal uterine cavity and prevent postoperative IUA (Han et al., 2016).

Secondly, hydrogels applied in IUA treatment should have controllable release profiles. As a simple and efficient platform for controlled release and delivery, the ideal bioactive hydrogels embed growth factors, drugs, and stem cells with therapeutic functions within their 3D structure. These drugs are released through the pores, hydrolysis of bonds, or self-degradation of the hydrogels, allowing the drugs to be applied to the uterine cavity wound after surgery with a sustained profile to promote regeneration of the endometrium (Sheth et al., 2019). Chemically crosslinked hydrogels usually provide a stable polymer network with slow degradation kinetics. For patients with severe IUA, where there is almost no normal functional endometrial tissue in the uterine cavity, stem cell transplantation is a potential option for the promotion of endometrial regeneration. Under such circumstances, the ideal bioactive hydrogels need to create a 3D cell delivery and culture system that is conducive to transplanted stem cell survival, proliferation, and directed differentiation (Cervelló et al., 2015).

Depending on the source, hydrogels can be divided into natural hydrogels and synthetic hydrogels. Natural hydrogels (e.g., HA, alginate, chitosan, collagen, and polyethylene glycol) have already been widely used in the field of regenerative medicine (Liu et al., 2020a). They are generally highly biocompatible, as reflected in their successful use in the peritoneum and other sites *in vivo* (T et al., 2016). Their biocompatibility is promoted by a high-water content and a physiochemical similarity to the native extracellular matrix, both compositionally (particularly in the case of carbohydrate-based hydrogels) and mechanically (Larrañeta et al., 2018). The naturally derived materials often have desirable biological properties and can influence cell function, but some natural hydrogels are limited by poor mechanical strength and fast degradation profiles (Rastogi and Kandasubramanian, 2019). In contrast, synthetic polymers provide appropriate 3D environments and have the desired mechanical strengths. However, they lack the bioactive properties of natural materials. Therefore, it is necessary to produce hybrid materials by combining synthetic and natural polymers, and to retain the desirable characteristics of both materials (Liu et al., 2018).

To date, hydrogels used in clinical settings can only achieve physical anti-adhesion. Bioactive hydrogels with controlled release and suitable culture microenvironment have not been widely used in the treatment of IUA. Hydrogels have been widely exploited with the aim of achieving drug delivery directly into the vaginal mucosa for hormone therapy, owing to their attractive features such as the increased drug residence time at the targeted sites and control of drug release rates after hysteroscopic transcervical resection of adhesion (TCRA) (Shamloo et al., 2018).

## The Application of Bioactive Hydrogels in IUA

The excellent biocompatibility, controllable mechanical properties, the ability of incorporating therapeutic agents for sustained release, and encapsulating seed cells at physiological conditions make bioactive hydrogel an ideal option for IUA treatment and endometrial regeneration. Several factors should be considered carefully, such as vascularization, native cell recruitment, and scar inhibition (Owusu-Akyaw et al., 2019). In the field of IUA treatment, hydrogels are broadly applied as space-filling agents, delivery vehicles for bioactive substances, and 3D structures that organize cells and present stimuli to ensure the repair of the damaged endometrium (Grover et al., 2012). Herein, we summarize the progress in hydrogel research for endometrial regeneration and IUA treatment from the following three aspects; namely, bioactive hydrogels as physical anti-adhesion barriers, *in situ* drug delivery systems, and 3D cell delivery and culture systems.

### Bioactive Hydrogels as Absorbable Physical Anti-Adhesion Barriers in IUA

Generally speaking, the main methods for the treatment of IUA are transabdominal or hysteroscopic resection of the adhesion; but while the former creates appreciable tissue injury, the latter has a high risk of recurrence, and postoperative uterine cavity wounds often quickly form new adhesions (Bhandari et al., 2015). Therefore, positive preventive measures should be taken for IUA after operation. Currently, strategies to reduce the rate of recurrence and improve reproductive outcomes in cases of severe IUA include the use of an IUD, Foley balloon (FB), amnion graft applied over a FB, and an oxidized and regenerated cellulose adhesion barrier (INTERCEED^®^), with the aim of maintaining the uterine lumen (Ugboaja et al., 2017). However, there are several shortcomings of IUD and FB in preventing the recurrence of IUA. IUD has a potential risk factor of infection (Sun et al., 2018a), and is only effective in preventing re-adhesion (Chi et al., 2018). To solve the problem, some studies investigated the potential benefits of hydrogels as physical anti-adhesion barriers to prevent recurrence in IUA after hysteroscopic adhesiolysis (Table 1).

**TABLE 1:** Summary of bioactive hydrogels as physical barriers used in IUA.

Physical barrier	Biomaterial	Model	Strength	References
IUD + Hydrogel	New crosslinked hyaluronan gel	Human	The application of new crosslinked hyaluronan gel significantly reduces IUA formation	Li et al. (2019)
Hydrogel	New crosslinked hyaluronan gel	Human	New crosslinked hyaluronan gel appears to be able to reduce the formation of IUA	Can et al. (2018)
IUD + Hydrogel	New crosslinked hyaluronan gel	Human	Better endometrial thickness values were observed in those who received new crosslinked hyaluronan gel either alone or in combination with an IUD	Pabuçcu et al. (2019)
Hydrogel	ABT13107	Human	ABT13107 is non-inferior to the highly viscous hyaluronic acid anti-adhesive barrier in IUA formation after hysteroscopic surgery	Lee et al. (2020)
FB + Hydrogel	Auto-crosslinked hyaluronic acid gel	Human	Auto-crosslinked HA gel could be able to reduce IUA, decrease adhesion severity, and improve menopause postoperatively	Xiao et al. (2015)
Hydrogel	Hyaluronic acid gel	Human	Reduce the risk of postoperative IUA	Tafti et al. (2021)

Compared with patients using an IUD alone, the combination of an oxidized, regenerated cellulose adhesion barrier (INTERCEED^®^) and an IUD could regain an adhesion-free uterine cavity and significantly shorten the conception interval (Cai et al., 2017). A comprehensive treatment strategy of intrauterine injection of HA hydrogel combined with a balloon urinary catheter and IUD insertion, and oral estrogen following hysteroscopic adhesiolysis, was investigated in moderate-severe IUA patients (Xiong et al., 2020). The results indicated that this combined strategy prevented the recurrence of IUA to a certain extent. Moreover, evidence indicated that the combination of IUD and anti-adhesion hydrogel had a more satisfactory effect than applying an IUD alone (Can et al., 2018; Li et al., 2019). Lin *et al.* (Lin et al., 2013) found that the insertion of an intrauterine balloon or IUD was more effective than the use of HA gel alone in the prevention of IUA reformation. Furthermore, Pabuçcu *et al.* (Pabuçcu et al., 2019) found that endometrial thickness was significantly enhanced when applying a new crosslinked hyaluronan gel with an IUD after hysteroscopic adhesiolysis. Although many studies have shown that an IUD combined with hydrogels can reduce IUA recurrence, in some cases the clinical pregnancy rate of these patients is not improved. Therefore, in-depth research is needed to explore whether the application of hydrogels can improve pregnancy outcomes for infertile patients. It may be that the pure hydrogels only play an anti-adhesion role, and are unable to promote the regeneration and functional recovery of the endometrium. Therefore, it is necessary to optimize the design to prepare functional bioactive hydrogels that are conducive to endometrial repair and functional regeneration of the endometrium.

Huang *et al.* (Huang et al., 2020b) tested patented intrauterine scaffolds made of amino acids as a barrier in the treatment of recurrent IUA with poor prognosis. The results showed that the menstrual volume returned to normal, and the recovery rate of amenorrhea increased significantly after scaffold implantation. However, the recurrence rate of IUA was still up to 25%, which indicated that although intrauterine scaffolds may be an effective therapy, they still need to be further optimized.

In addition, although ordinary HA gel can mechanically support the uterine cavity, because of its rapid degradation it cannot stay inside the uterine cavity for a prolonged duration, therefore the anti-adhesion effect is not satisfactory (Azumaguchi et al., 2019). A meta-analysis addressing the use of HA gel to prevent IUA after intrauterine surgery showed that HA gel can reduce the incidence of re-adhesion in mild cases following intrauterine surgery (Fei et al., 2020; Zheng et al., 2020). While there was also research showed that the application of auto-cross-linked HA gel was unable to reduce the recurrence rate of IUA following hysteroscopic adhesiolysis (Zhou et al., 2021). ABT13107, a newly developed thermo-responsive sol-gel made from non-animal derived HA and synthetic poloxamers, had been studied regarding preventing the recurrence of IUA (Lee et al., 2020). Moreover, a randomized double-blind controlled clinical trial (RCT) was conducted to evaluate the efficacy and safety of auto-crosslinked HA gel for preventing IUA after hysteroscopic adhesiolysis; the results indicated that auto-crosslinked HA gel may be able to reduce IUA, decrease adhesion severity, and improve menopause postoperatively (Xiao et al., 2015). A meta-analysis exploring whether the application of HA gel can reduce the recurrence rate of IUA after hysteroscopic adhesiolysis showed that HA gel could reduce the recurrence rate of IUA, but had no significant effect on postoperative pregnancy rate (Fei et al., 2019). In addition, another study compared the effect of adjuvant therapy on the prevention and treatment of IUA and found that an alginate hyaluronate-carboxymethylcellulose membrane (ACH) was most effective in preventing IUA progression (Yan and Xu, 2018). Chenite *et al.* indicated that sodium hyaluronate or a hydrogel prepared using hydroxybutyl chitosan (HBC) and polyphosphate can reduce the occurrence of postoperative IUA. However, owing to the lack of viscosity of sodium hyaluronate or HBC hydrogel, they are not easily retained in the uterine cavity. Thus, it cannot completely isolate uterine wounds, which affects its ability to prevent IUA. Therefore, after developing and optimizing hydrogels with potential anti-adhesion ability combined with a physical anti-adhesion barrier for IUA, bioactive hydrogels overcome some drawbacks of original natural hydrogels, the prevention and treatment have great clinical significance (Kowalski et al., 2019).

### Bioactive Hydrogels as *in situ* Drug Delivery Systems for Endometrial Regeneration

With the development of tissue engineering, it is possible to use biomaterials to repair and replace damaged tissues and organs (Kwon et al., 2018). The application of bioactive hydrogels in IUA endometrial tissue regeneration is still in the exploratory stage. However, the use of bioactive hydrogels as a local drug delivery system to release specific drugs for regulating the specific pathological environment of IUA has great potential for application in endometrial regeneration. Hydrogels have attracted noticeable interest for their use in drug delivery owing to their unique physical properties. The high porosity that characterizes hydrogels can easily be adjusted by controlling the density of cross-links in their matrix and their affinity to water (Ávila-Salas et al., 2019). Their porous structure also allows drugs to be loaded and then released. The topical application of hydrogels can effectively be used in drug delivery that can help to alleviate the symptoms of many pathological conditions. *In situ* drug delivery systems have become a popular treatment owing to their combination of a physical barrier and controlled drug release. Hydrogels made of hydrophilic polymers with large amounts of water are suitable for drug delivery when applied to IUA patients. Drugs such as β-estradiol can be delivered by hydrogel scaffolds for various purposes including endometrial regeneration (Lam et al., 2014). Furthermore, the use of stimuli-responsive hydrogels brings many possibilities in drug delivery systems.

#### Bioactive Hydrogels as *in situ* Drug Delivery Systems Through Vaginal Administration

The large surface area and abundant blood flow in the vagina are excellent for promoting drug absorption (Ci et al., 2017). Vaginal administration has no first-pass effect and has low enzyme activity; therefore, drugs can perform local treatment, and can also enter the systemic circulation. Thermosensitive hydrogels are easily administered vaginally, and are administered in a low viscous form at room temperature (Domiński et al., 2019). After entering the vagina, the hydrogel spreads rapidly into the folded area of the vaginal mucosa and forms a gel at body temperature (Liu et al., 2017). Stromal cell-derived factor-1α (SDF-1α) is a chemokine protein with the ability of recruiting endogenous cells, that can accelerate the regeneration of multiple tissues. In order to obtain a superior effect, Qi *et al.* (Wenbo et al., 2020) prepared an SDF-1α-loaded-chitosan-heparin hydrogel with controlled drug release ability. Seven days after treatment, uteri treated with SDF-1α incorporating a hydrogel gave both mechanical support to the uterine cavity and also initiated regeneration of the endometrium by recruiting c-kit positive stem cells to the injury site. Cai *et al.* (Cai et al., 2019) studied the incorporation of SDF-1α within a silk fibroin-bacterial cellulose (SF-BC) membrane that promoted the regeneration of a full-depth uterine injury and also improved the pregnancy outcome of the damaged uterus.

**FIGURE 1:**
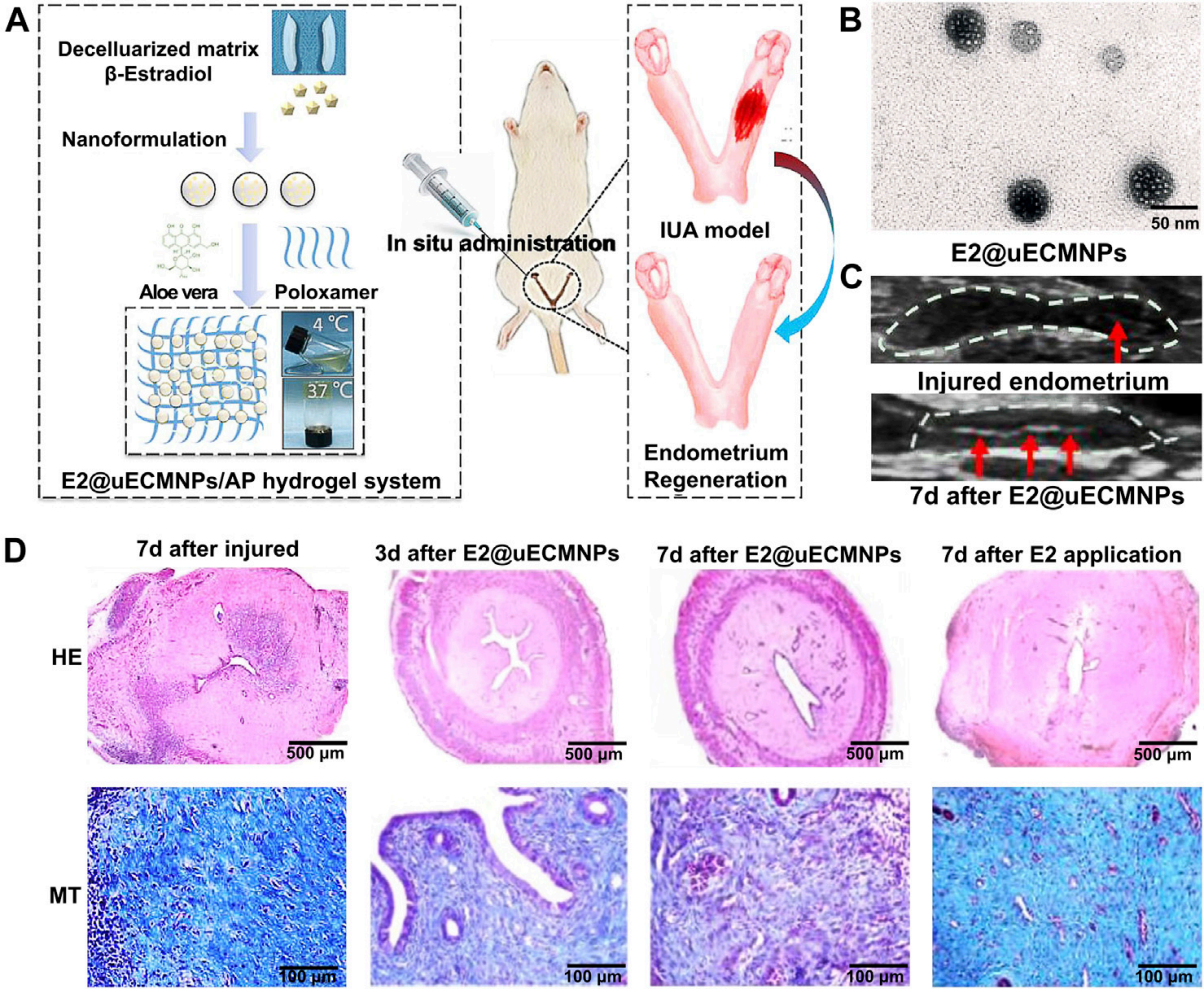
An overview of E2@uECMNPs/AP hydrogel system with multi-therapeutic effects to promote endometrial regeneration for the prevention of IUA. **(A)** Schematic graphs of E2@uECMNPs/AP hydrogel system. **(B)** TEM images of E2@uECMNPs. **(C)** Comparison of ultrasound images of the injured endometrium with or without E2@uECMNPs treatment. **(D)** H&E staining (HE) and Masson staining (MT) of the uterus from IUA rat’s uteri and IUA rats uteri receiving different treatments on day 3 and 7 after injury. Reproduced with permission (Yao et al., 2020). Copyright 2020, Elsevier Ltd.

Aloe has been reported as an ideal organic component to mix with poloxamer to form a more biologically friendly thermosensitive hydrogel system (Baghersad et al., 2018). Aloe-poloxamer (AP) hybrid hydrogel has been fabricated for treating IUA, which can achieve a better therapeutic effect with prolonged retention time within the uterine cavity compared to poloxamer gel. This study showed that AP hydrogel can serve as a biologically active delivery carrier for β-estradiol and that it can synergistically promote morphological, structural, and functional repair of the injured uterus (Yao et al., 2020) (Figures 1A–D). The E2@uECMNPs/AP hydrogel itself acts as a physical barrier during the early stage of the injury. Its multiple components, including β-estradiol, synergistically exert pro-healing effects and promote endometrial regeneration. From the perspective of anti-adhesion therapy and sustained drug release, hydrogels have potential implications for vaginal administration in the treatment of IUA.

#### Drug Delivery Through Injectable Bioactive Hydrogels

The use of injectable hydrogels, where the solution–gel transition occurs at the site of administration, may shorten the duration of treatment, reduce the risk of infection of the implanted site, and prevent scarring (Asai et al., 2012). To date, various studies have investigated the possibility of this therapy in animal models (Sun et al., 2018b; Jian et al., 2018; Jiang et al., 2019). The injectable SDF-1α release hydrogel exhibited long-term recruiting of hematopoietic stem cells (HSCs) and achieved better effects than a one-off injection of SDF-1α solution. This hydrogel could be a candidate for uterine injury healing and other wound dressing drug delivery systems. Because of their biocompatibility, biodegradability, and tissue adhesion, chitosan and HA are also suitable as a matrix for injectable *in situ* hydrogels for drug, gene delivery, and tissue repair through invasive surgery (Lu et al., 2019).

**FIGURE 2:**
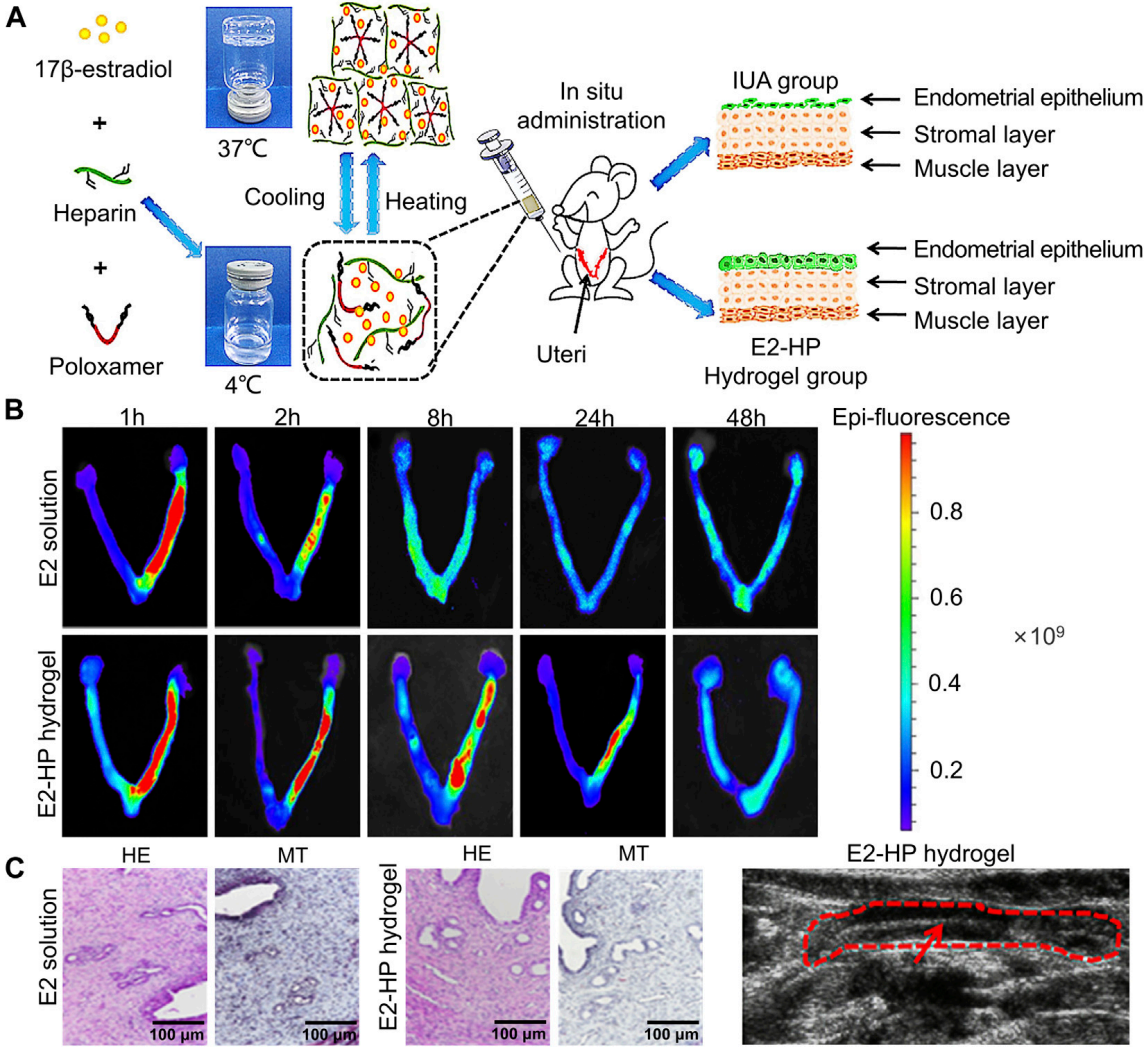
Construction of E2-HP hydrogel and *in-situ* administration effect. **(A)** Schematic diagram of E2-HP hydrogel as an *in-situ* administration drug for the treatment of IUA. **(B)** The penetration and retention of different FITC-E2 formulations in the uterus after *in-situ* administration. **(C)** Comparison of H&E staining (HE) and Masson’s staining (MT) of endometrium at 14 days after different treatments. **(D)** Ultrasound images of the endometrium after E2-HP hydrogel treatment. Reproduced with permission (Zhang et al., 2017). Copyright 2021 Dove Press Ltd.

**FIGURE 3:**
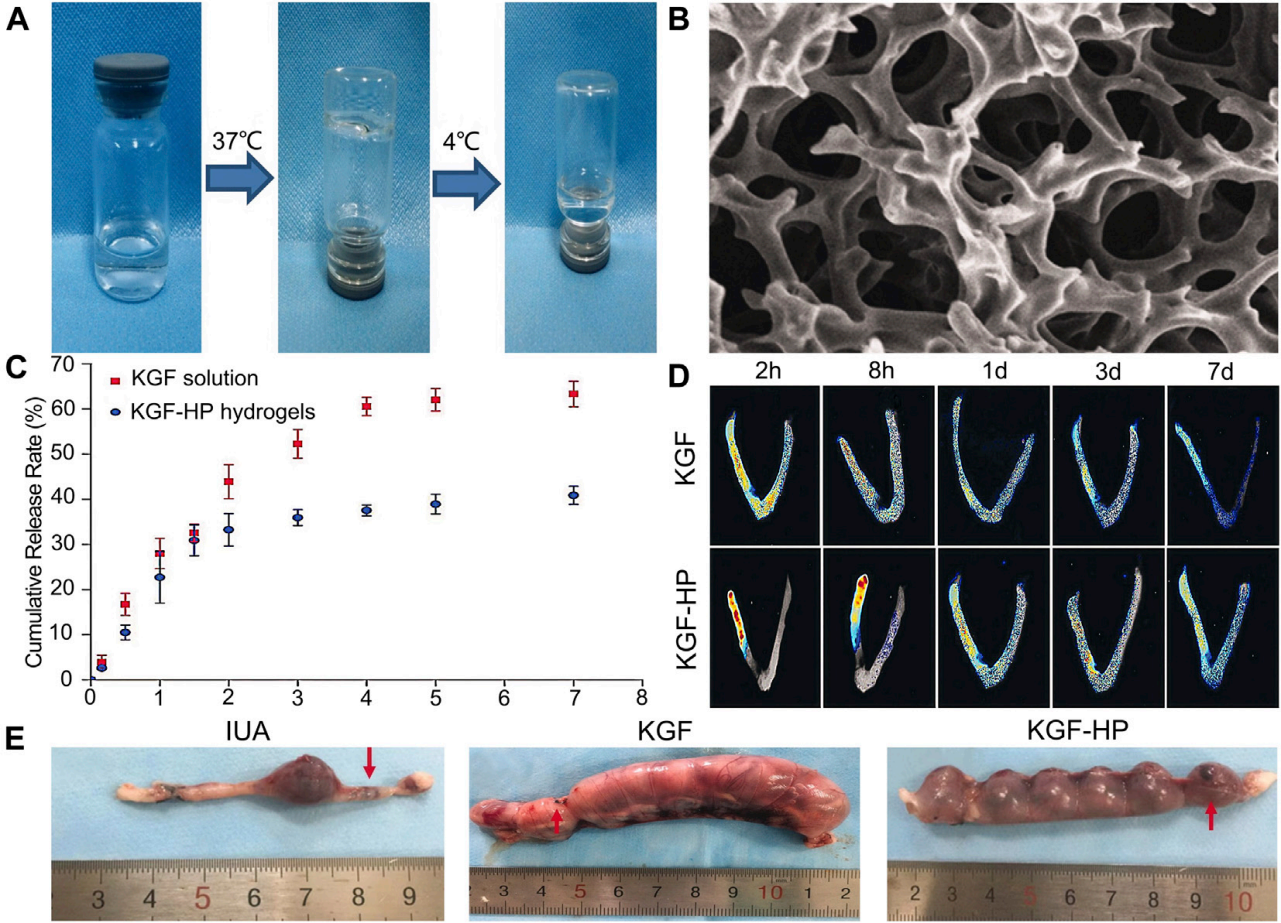
Construction of KGF-loaded HP hydrogel and its curative effect. **(A)** The temperature-dependent appearance of KGF-loaded HP hydrogel. **(B)** SEM images of the lyophilized KGF-loaded HP hydrogel. **(C)** The cumulative release profile of free KGF solution and KGF-HP hydrogel. **(D)** Representative fluorescence images of an intact rat uteri after treatment with FITC-labeled KGF or KGF-loaded HP hydrogel. **(E)** Representative images of embryo implantation for rats treated by different formulations. Reproduced with permission (Xu et al., 2017). Copyright 2017 Taylor & Francis Group.

The role of new bioactive hydrogel scaffolds, such as heparin-poloxamer (HP), and gelatin meth acryloyl (GelMA), in uterine cavity repair has attracted growing attention, and they can be used as sustained-release drug delivery systems to effectively promote endometrial repair in IUA animal models (Wu et al., 2019). Zhang *et al.* (Zhang et al., 2017) injected the combination of different drugs (hormone or growth factor) with thermosensitive, sustained drug delivery systems based on HP hydrogels to rat’s uteri (Figures 2A–D). The study demonstrated that the encapsulation of 17 β-estradiol into HP hydrogels can increase the drug concentration at the injured area while reducing the blood concentration, because it can be administered by *in situ* injection. Keratinocyte growth factor (KGF), a potent repair factor for epithelial tissues, had been encapsulated into HP hydrogels, and the KGF-loaded HP hydrogel delivered a sustained release of long duration and prolonged retention of the encapsulated KGF in the injured endometrium, thus improving endometrial recovery in a rat model compared to the administration of KGF alone (Figures 3A–E) (Xu et al., 2017). However, at present, the above synthetic biological scaffolds are generally expensive and have not been widely used in clinical settings. In animal experiments, most injectable bioactive hydrogels are administrated through the myometrium rather than the cervical canal. However, gynecologists usually apply anti-adhesion hydrogel through the cervical canal for IUA patients after hysteroscopic adhesiolysis. Though these hydrogels are all injectable, different administrations make it is not clear whether these patients can benefit from injectable bioactive hydrogels.

### Bioactive Hydrogels as 3D Cell Delivery and Culture Systems in IUA

Stem cell therapies have been demonstrated to be promising and multifunctional alternatives to traditional drug therapy. To date, numerous maladies have been modeled in animals to test the efficacy and safety of a range of cellular therapies. However, the difficulty lies in the normal expansion of stem cells in cell therapy. There are two crucial factors that influence stem cell expansion, which are biochemical composition and the physical properties of the matrix (Madl et al., 2018). One crucial physical regulator of stem cell fate is matrix stiffness. Interestingly, bioactive hydrogels can be fabricated as culture systems with a tunable stiffness that encompasses a physiological range. Owing to their high-water content, porosity, and soft consistency, bioactive hydrogels can closely simulate natural living tissue. In addition, by adjusting the biochemical composition of the hydrogel or encapsulated drugs, they can also regulate the fate of stem cells. Therefore, functional bioactive hydrogels make it easier to transport stem cells to injured the endometrium with good vitality and differentiation potential. At present, there are ongoing studies on the advantages and disadvantages of different bioactive hydrogels applied in stem cell therapies in IUA. A summary of bioactive hydrogels in stem cell therapy in IUA is listed. The advantages of the mentioned hydrogels are also listed (Table 2). According to Table 2, the endometrium regeneration associated molecules are well improved. Another highlight of bioactive hydrogel used in stem cell therapy is that it can decrease uterine fibrosis rate and promote endometrial glands growth.

**TABLE 2:** Summary of bioactive hydrogels in cell therapy in IUA.

Cell	Biomaterial	Model	Molecule/pathway	Strength	References
-	Chitosan-heparin hydrogels	Sprague-Dawley rats	Downregulated expression of TGF-β	SDF-1α/CXCR4 axle secreted cytokine to regulate the tissue regeneration	Wenbo et al. (2020)
			Upregulated expression of VEGF		
-	E2@uECMNPs/AP hydrogel	Sprague-Dawley rats	Upregulated Ki67, cytokeratin, and ER-β	Increased morphological recovery and decreased uterine fibrosis rate	Yao et al. (2020)
			Decreased TGF-β1 and TNF-α		
-	E2-Heparin-Poloxamer Hydrogel	Sprague-Dawley rats	Kisspeptin; ERK1/2 and MAPKs p38 pathways	Facilitate the regeneration	Zhang et al. (2020b)
				Inhibiting the cell apoptosis in IUA model	
dEMSCs	Hyaluronic acid fibrinogen/thrombin hydrogel (HA + F + T50 gel)	Sprague-Dawley rats	Increased expression of PECAM and IGF-1	Expression and secretion of molecules essential for embryonic implantation	Kim et al. (2019)
			VEGF and LIF expression are also increased	Shortens time window between cell transplantation and embryo transfer	
MSC-Sec	Crosslinked hyaluronic acid gel	Sprague-Dawley rats	-	Creates a sustained release system	Liu et al. (2019)
				Thicker endometrium and more glands were observed after treatment	
BMSCs	Pluronic F-127-vitminC hydrogel	Sprague-Dawley rats	Restored cytokeratin, von Willebrand Factor (vWF)	Vc promoted the survival and health of PF-127-encapsulated BMSCs *in vitro*	Yang et al. (2017)
			Decreased interleukin-1β (IL-1β)	Thicker endometrium membrane	
				More glands and fewer areas of fibrosis	
Oral mucosal epithelial cells	Lyophilized amniotic membrane	Sprague-Dawley rats	VEGF expression is increased	Effective in preventing fibrosis with improved regeneration of endometrium and endometrial glands in the rat model of IUA	Chen et al. (2019)
OMECs					
hUCMSC	Collagen	Sprague-Dawley rats	Increased the expression of HuNu and vimentin in a IUA-induced rat model	Increased number of endometrial glands and reduced area of fibrosis	Liu et al. (2020b)
			Protein levels of the p-transcriptional co-activator with PDZ-binding motif, stromal cell-derived factor-1, and C-X-C chemokine receptor type 4 were upregulated		
UC-MSCs	Collagen	Human	-	38.4% participants had a successful pregnancy	Cao et al. (2018)
UC-MSC-derived exosomes	Collagen	Sprague-Dawley rats	Increased the expression of the estrogen receptor α/progesterone receptor	Endometrium regeneration	Xin et al. (2020)
				Collagen remodeling	
				Increased the expression of the estrogen receptor α/progesterone receptor	
				Restored fertility	
				Reduced inflammation, and increased anti-inflammatory responses	
hiMSCs	Alginate and gelatin	Sprague-Dawley rats	Integrin αv β3 and LIF were significantly higher in the cell-loaded scaffold group	Promoted the recovery of the endometrial histomorphology (endometrial tissue and gland regeneration) and the regeneration of endometrial cells (stromal cells and epithelial cells) and endothelial cells	Ji et al. (2020)
				Improved endometrial receptivity functional indicators	
ADSCs-derived exosomes	PEG	Sprague-Dawley rats	Significant increases in VEGF, LIF, avβ3, and IGF-1 expression	Promote HUVEC proliferation, migration, and tube formation *in vitro*	Pan et al. (2020)
				Exhibited a robust ability to induce neovascularization and tissue regeneration while suppressing localized fibrosis *in vivo*	
				Regenerated endometrial tissue was more receptive to embryos, leading to higher rates of pregnancy	

hAECs: human amnion epithelial cells; bFGF: basic fibroblast growth factor; VEGF: vascular endothelial growth factor; IGF-1: insulin-like growth factor-1; COL1A1: collagen type I alpha 1; TIMP-1: tissue inhibitor of metalloproteinase-1; TGF-β: transforming growth factor-β; PDGF-C: platelet-derived growth factor-C; THBS1: thrombospondin-1; CTGF: connective tissue growth factor; ER-β: estrogen receptor β; E2@uECMNPs/AP hydrogel: nanoparticulate decellularized uterus (uECMNPs) encapsulated β-estradiol thermosensitive aloe-poloxamer hydrogel; MenSCs: menstrual blood stem cells; BMSCs: bone marrow-derived stem cells; dEMSCs: decidualized endometrial stromal cells; MSC-Sec: mesenchymal stem cell-secretome; UC-MSCs: umbilical cord-derived mesenchymal stromal cells; ADSCs: adipose stem cells.

#### Hyaluronic Acid

HA may be an appropriate hydrogel for endometrial regeneration therapeutics. Nevertheless, HA gels have a natural half-life of just 1–2 days (Carruthers and Carruthers, 2007). Moreover, the half-life may be even shorter in the uterus owing to aqueous dilution. In order to optimize the short half-life of natural HA, Kim *et al.* (Kim et al., 2019) constructed endometrium-tailored HA/fibrin composite hydrogel and demonstrated the significant regenerative effects of the *in vitro* processed isotopic cells encapsulated in composite scaffold materials. The expression of *PECAM* and *IGF-1*, which are neovascularization- and decidua-specific genes, were significantly increased. Vascular endothelial growth factor (VEGF) and leukemia inhibitory factor (LIF) expression were also increased in the HA-fibrin-thrombin 50 mg (HA-F-T50) composite hydrogel. Moreover, an attempt at pregnancy was possible as early as 2 weeks after treatment, and successful *in vivo* embryonic implantation and development in the regenerated recipient model was confirmed at 7 days after embryo transformation in a murine uterine synechiae model. Despite limited evidence of its efficiency in the human body, further studies should be carried out to optimize this promising technology.

**FIGURE 4:**
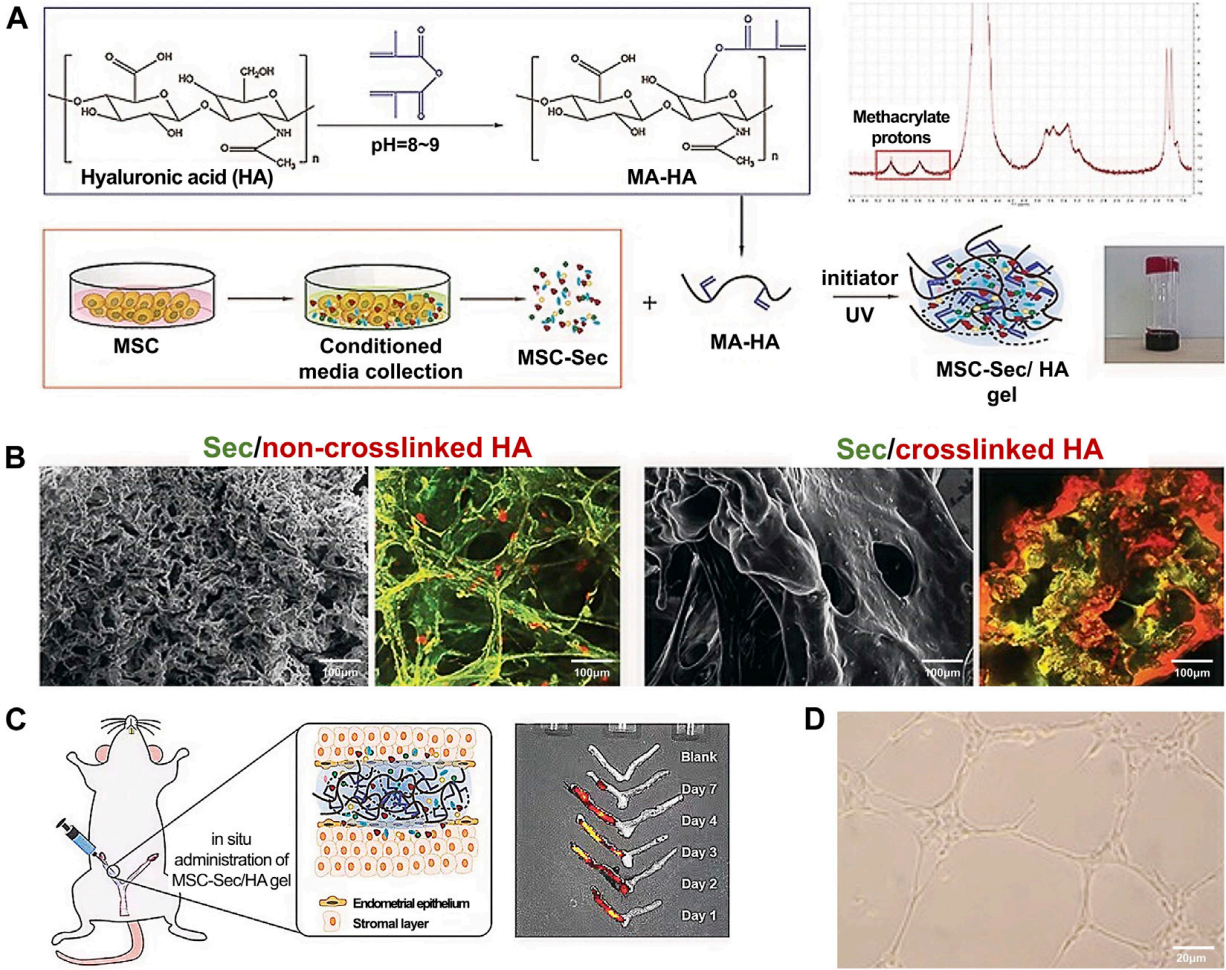
Fabrication and characterization of MSC-Sec-loaded crosslinked HA gel. **(A)** Schematic showing the synthesis of MSC-Sec-loaded, crosslinked HA gel and the state of HA gel at room temperature when the bottle is upside down. **(B)** Representative SEM and color-depth projection confocal images of MSC-Sec/non-crosslinked HA and MSC-Sec/crosslinked HA; green represents MSC-Sec, red represents HA. **(C)** MSC-Sec/HA gel injection and a rodent model of endometrial injury. **(D)** The tube-formation ability of human umbilical vein endothelial cells is enhanced in the MSC-Sec-treated group. Reproduced with permission (Liu et al., 2019). Copyright 2019 WILEY-VCH Verlag GmbH & Co. KGaA, Weinheim.

Liu *et al.* (Liu et al., 2019) fabricated a control-released, intrauterine-administered mesenchymal stem cell-secretome (MSC-Sec)-loaded, crosslinked HA gel, that was developed as a therapy to restore injured endometrial morphology and fertility in a rat model of AS (Figures 4A–D). This crosslinked HA gel can be used in stem cell therapy because it can bypass the tumorigenic risks associated with stem cell therapies and help maintain the promising effect of MSC-Sec on endometrial and endothelial cells.

#### Other Hydrogels

Apart from HA hydrogel, Pluronic F-127 is a synthetic Food and Drug Administration (FDA)-approved compound which has several advantages, including low toxicity, biocompatibility, and thermo-reversibility. It is widely used in drug delivery and *in vivo* tissue engineering because it can form into a hydrogel at physiological temperatures. BMSCs and Vitamin C encapsulated Pluronic F-127 hydrogel was prepared to promote restoration of damaged IUA endometrium *in vivo*; the vitamin C promoted BMSCs survival and growth in the hydrogel (Yang et al., 2017).

In another study, Liu *et al.* (Liu et al., 2020b) used collagen scaffold loading with human umbilical cord mesenchymal stem cells (hUCMSCs) to increase the number of endometrial glands and reduced the area of fibrosis, suggesting that the combination of the collagen scaffold and hUCMSCs may be an alternative approach for treating IUA. Moreover, the hUCMSCs-collagen scaffold had passed through a phase I clinical trial and obtained a satisfactory outcome, where around 38.4% of participants underwent a successful pregnancy without complications associated with stem cell therapy (Cao et al., 2018).

**FIGURE 5:**
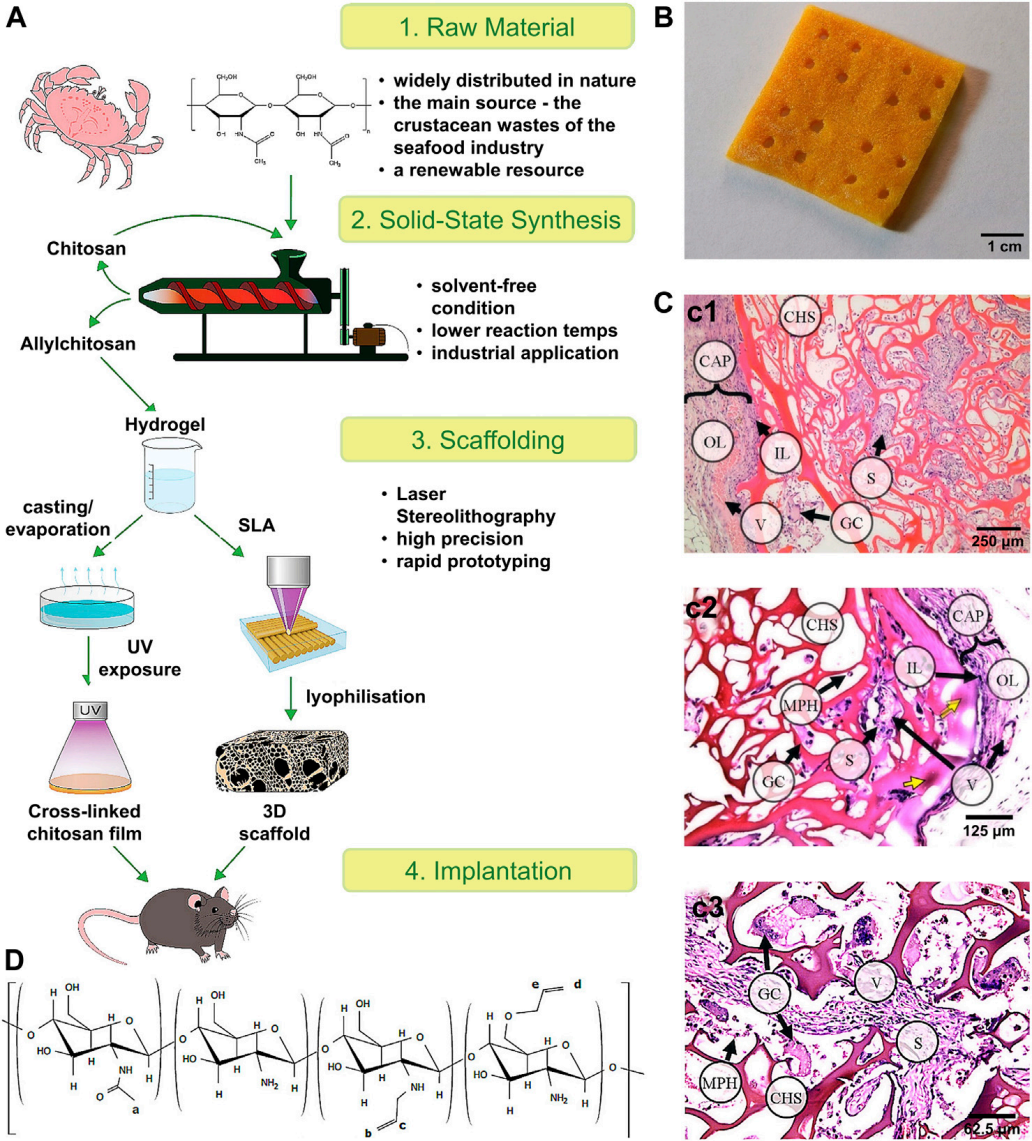
Scheme of the experimental work and the tissue reaction to the 3D scaffold. **(A)** Scheme of the experimental work. **(B)** 3D porous allylchitosans scaffold (the chitosan sponges) after freeze drying. **(C)** Tissue reaction to the porous 3D scaffolds based on allylchitosans. c1) 30 days: the chitosan sponge (CHS) material was oxyphilic; c2) 60 days: a basophilic focus (yellow arrows) in the surface septa of the CHS material; and c3) 90 days: deep CHS sections: most of the scaffold septa were moderately basophilic. **(D)** The structure of the synthesized allylchitosans. Reproduced with permission (Bardakova et al., 2019). Copyright 2021, Elsevier Ltd.

**FIGURE 6:**
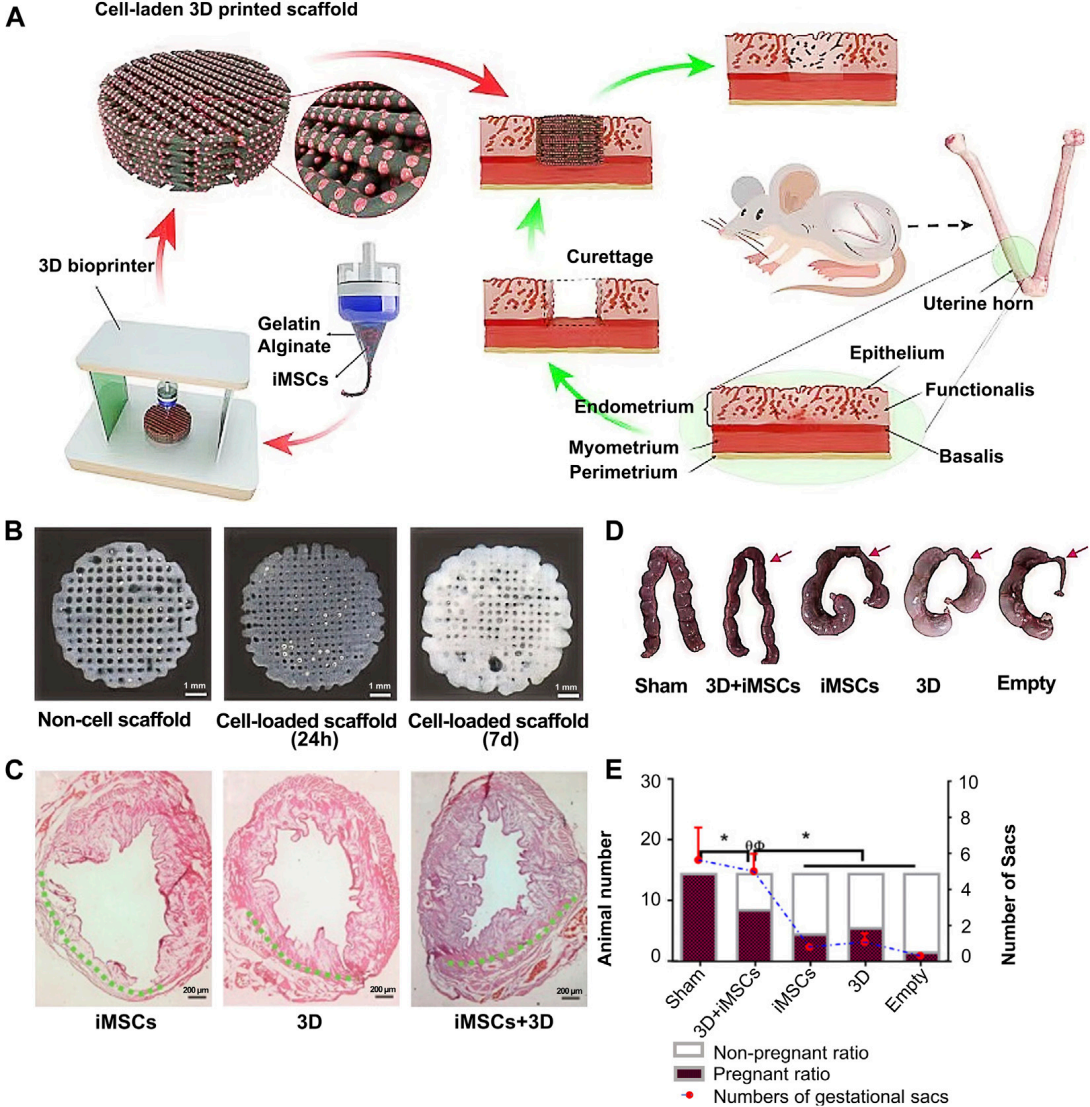
Whole overview of 3D bioprinting a human iPSC-derived MSC-loaded scaffold. **(A)** The whole preparation procedure of an alginate–gelatin hydrogel scaffold and scaffold implantation. **(B)** Picture of the original alginate-gelatin hydrogel and cell-loaded scaffold at 24 h and 7 d **(C)** H&E stain of the endometrium after cell implantation, scaffold implantation, and cell-loaded scaffold implantation. **(D–E)** Outcome of endometrial receptivity after cell-loaded scaffold implantation. Reproduced with permission (Ji et al., 2020). Copyright 2021, Elsevier Ltd.

In terms of 3D bioactive hydrogel scaffolds, chitosan is a biopolymer with a unique set of biological and physicochemical properties (Pan et al., 2020). Thus, Ksenia *et al.* (Carruthers and Carruthers, 2007) successfully formed different scale structures of chitosan and further tested their biomechanical quality and biocompatibility to demonstrate the prospects of their broad biological application (Figures 5A–D). Furthermore, Ji *et al.* (Ji et al., 2020) studied the application of 3D bioprinting of a human iPSC-derived MSC-loaded scaffold for repair of the uterine endometrium. The results showed that the endothelial cells and endometrial cells regenerated, and the dysfunctional endometrium was partially restored (Figures 6A–E).

**FIGURE 7:**
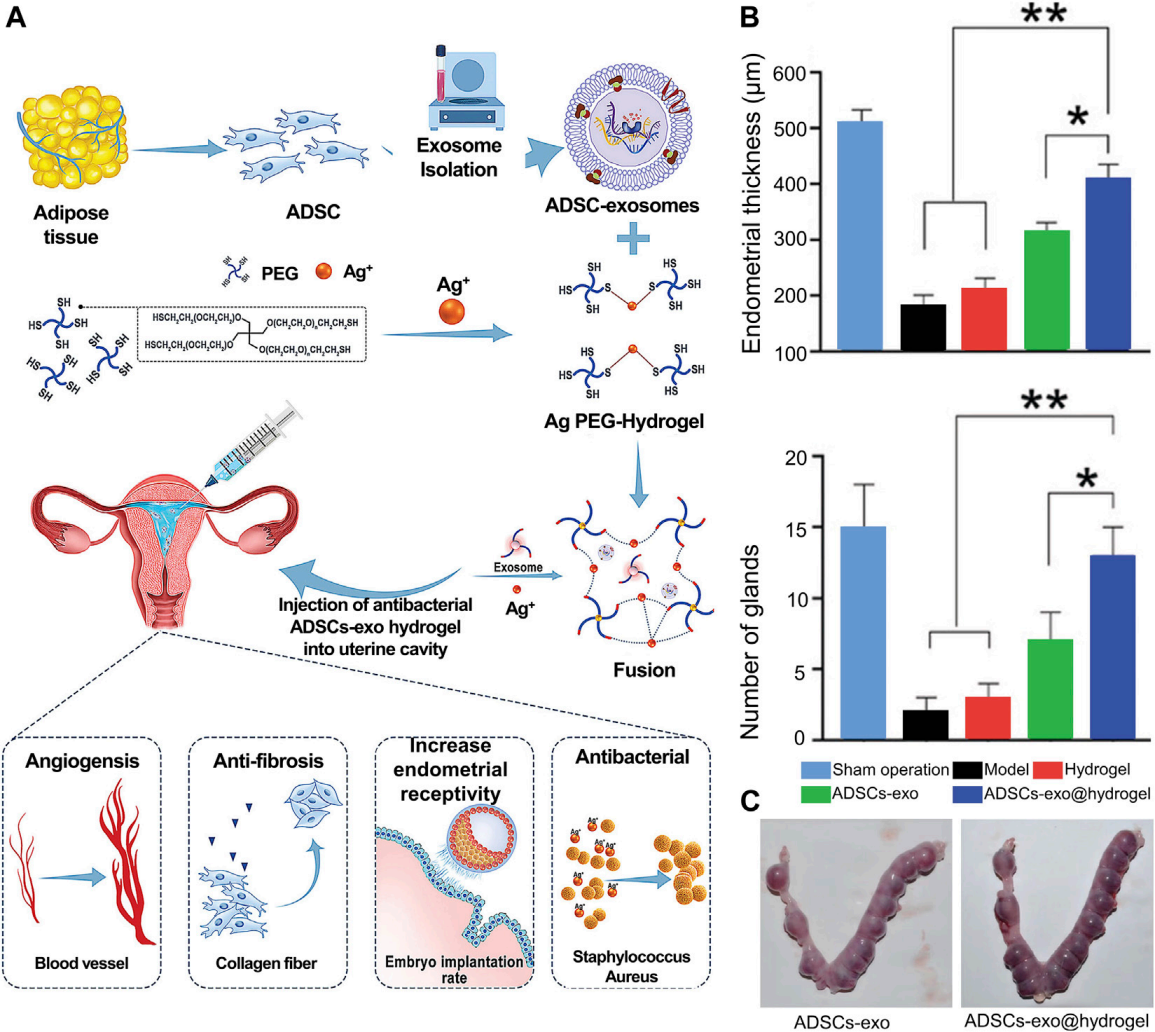
Whole overview of ADSC-exosome-hydrogel. **(A)** Preparation procedures of ADSCs-exosome hydrogel. **(B)** Performance of ADSC-exosome-hydrogel in endometrial regeneration. **(C)** Performance of ADSC-exosome-hydrogel in endometrial receptivity. Reproduced with permission (Lin et al., 2021). Copyright 2021 WILEY-VCH Verlag GmbH & Co. KGaA, Weinheim.

Recently, bioactive hydrogels laden with stem cell exosomes have been developed for endometrial regeneration. The advantages of hydrogel treatment include the potential for sustained exosome release, resulting in the maintenance of higher local concentrations of pharmacologically important compounds for extended periods, and reducing the need for repeated dosing in a clinical setting, thus making them a promising candidate for use in exosome-based endometrial repair applications (Pan et al., 2020). Lin *et al.* (Lin et al., 2021) developed a microenvironment-protected exosome-hydrogel for endometrial regeneration (Figures 7A–C). This PEG hydrogel was injectable, antibacterial, and facilitated controlled ADSC-exo release in order to promote endometrial regeneration.

## Current Limitations and Future Perspectives

Despite their many advantageous properties, hydrogels also have several limitations. With the continuous progress of related research, the disadvantages of natural hydrogels are being gradually revealed. Although some natural hydrogels such as HA and alginate have already shown superiority over other hydrogels, the opportunity to improve current hydrogel implementations is still very promising. Researchers are gradually improving the structure of hydrogels by changing the polymer type and optimizing fabrication methods. The low tensile strength of many hydrogels limits their use in load-bearing applications, resulting in displacement of the hydrogel from a targeted local site. Because the hydrophobic compounds have limited loading quantity and homogeneity in hydrogel matrices, hydrogels are limited to carrying hydrophilic drugs (Zagórska-Dziok and Sobczak, 2020).

### Physical Anti-Adhesion Barriers

According to the published research (Du et al., 2020), anti-adhesion hydrogels are usually placed in the uterine cavity to prevent recurrence after operation. However, the effect of anti-adhesion hydrogels alone is less satisfactory than that of a combination of hydrogels and IUD. The reason why hydrogels cannot become the first-line therapy in endometrial regeneration is that most hydrogel applications in the prevention of IUA are still less satisfactory than a traditional IUD. Moreover, hydrogels do not have sufficient mechanical strength to shape a normal uterine cavity throughout a complete treatment. Unlike traditional IUD, some hydrogels in liquid are able to transform to sodium gel after entering uteri, which may help more efficient formation of the uterine cavity in various sizes. It is known that most IUA patients in childbearing age hope to improve fertility through IUA treatment. However, some bioactive hydrogels as physical barriers cannot assist in the formation of a normal functional endometrium for embryo implantation because they cannot meet the requirement of excellent cell culture system and sufficient mechanical strength. Therefore, future research needs to address the optimization of bioactive hydrogels as physical barriers to increase their strength in shaping a normal uterine cavity while still maintaining the capacity of controllable degradation.

### Drug Delivery Systems

As mentioned previously, hydrogels can be applied as *in situ* drug delivery systems for sustained release drugs to promote endometrial regeneration in patients with IUA. Compared with traditional hormone replacement therapy, this method may reduce the incidence of related risks like breast hyperplasia caused by oral estrogen in treating IUA. Several experiments that demonstrate that the effectiveness of a hydrogel-based drug delivery model in treating IUA have used animal models, and there has been limited evidence of clinical practice in human (Wang et al., 2020b). Whether hydrogels as *in situ* drug delivery systems are more beneficial than traditional drug delivery is still not clear in humans. Moreover, the hydrated nature of hydrogels can make terminal sterilization difficult and time-consuming (Lima et al., 2020). In the future, hydrogels with multiple concurrent functions may be a satisfactory way of treating IUA, which can provide both mechanical structure in forming the uterine cavity, and also be a stable *in situ* drug delivery system.

### Stem Cell Therapy

Because of their structural features, bioactive hydrogels are widely used as 3D cell culture systems. With the recent broader understanding that stem cells and stem cell derivatives can promote endometrial repair, the value of 3D hydrogel scaffolds that can simulate the living environment of cells and promote the transmission of stem cells and their secretions has become particularly prominent. Therefore, some studies have explored the application of hydrogels in IUA cell therapy. Many animal experiments have shown that, compared with traditional cell therapy, the application of hydrogels as a 3D culture environment produces superior outcomes in endometrial regeneration, and the improvement of endometrial receptivity is evident. However, despite exhibiting promising results in animal experiments, the application of hydrogels in stem cell therapy is still largely restricted to the experimental stage owing to its critical pitfalls and drawbacks such as safety issues, poor cell survival, and high cost.

Currently, one of the biggest concerns regarding stem cell therapy is the tumorigenic complications caused by uncontrollable stem cell implantation. Thus, the future study of hydrogels used in stem cell therapy could focus on controllable cell growth offering a better therapeutic approach while avoiding related complications. Moreover, the existing cell loaded hydrogels face the problem that the supply of oxygen and nutrients is limited by diffusion kinetics. For example, 3D printed hydrogels still require surface modification, such as chemical methods to carry integrin (for endometrial cells) and increase the survival rate of the seeded cells, by inducing cell-oriented differentiation, or by maturing the scaffold in a bioreactor before implantation (Wen et al., 2019). Therefore, selecting and optimizing bioactive hydrogels is important in obtaining better results and for their application in treating IUA.

## Conclusion

IUA is a common endometrial disease and one of the main causes of infertility in women of childbearing age. Owing to their excellent properties—such as good biocompatibility, degradability, and controlled drug release—bioactive hydrogels play an important role in the prevention and treatment of IUA and have great potential for application in the clinical setting. Bioactive hydrogels can be used as physical anti-adhesion barriers, and can also act as drug delivery systems for hormone drugs, multiple factors, and 3D cell delivery and culture systems in IUA treatment. These characteristics show that a combination of hydrogels and traditional intrauterine adhesion treatment can have a significant therapeutic effect on IUA and improve pregnancy success rates. While most researches ongoing are based on animal experiments, the mechanism behind the therapeutic effect of bioactive hydrogel in IUA treatment is not clear. The optimization on the safety and effectiveness of bioactive hydrogels is valuable to researchers in the future.

## References

[B1] Asai D. Xu D. Liu W. Garcia Quiroz F. Callahan D. J. Zalutsky M. R. (2012). Protein Polymer Hydrogels by *In Situ*, Rapid and Reversible Self-Gelation. Biomaterials 33 (21), 5451–5458. 10.1016/j.biomaterials.2012.03.083 22538198 PMC3801208

[B2] Ávila-Salas F. Marican A. Pinochet S. Carreño G. Valdés O. Venegas B. (2019). Film Dressings Based on Hydrogels: Simultaneous and Sustained-Release of Bioactive Compounds with Wound Healing Properties. Pharmaceutics 11 (9), 447. 10.3390/pharmaceutics11090447 PMC6781310 31480682

[B3] Azumaguchi A. Henmi H. Saito T. (2019). Efficacy of Silicone Sheet as a Personalized Barrier for Preventing Adhesion Reformation after Hysteroscopic Adhesiolysis of Intrauterine Adhesions. Reprod. Med. Biol. 18 (4), 378–383. 10.1002/rmb2.12294 31607798 PMC6780041

[B4] Baghersad S. Hajir Bahrami S. Mohammadi M. R. Mojtahedi M. R. M. Milan P. B. (2018). Development of Biodegradable Electrospun Gelatin/aloe-Vera/poly(ε-caprolactone) Hybrid Nanofibrous Scaffold for Application as Skin Substitutes. Mater. Sci. Eng. C 93, 367–379. 10.1016/j.msec.2018.08.020 30274069

[B5] Bardakova K. N. Akopova T. A. Kurkov A. V. Goncharuk G. P. Butnaru D. V. Burdukovskii V. F. (2019). From Aggregates to Porous Three-Dimensional Scaffolds through a Mechanochemical Approach to Design Photosensitive Chitosan Derivatives. Mar. Drugs 17 (1), 48. 10.3390/md17010048 PMC6356335 30634710

[B6] Bhandari S. Bhave P. Ganguly I. Baxi A. Agarwal P. (2015). Reproductive Outcome of Patients with Asherman's Syndrome: A SAIMS Experience. J. Reprod. Infertil 16 (4), 229–235. 27110522 PMC4819213

[B7] Cai H. Qiao L. Song K. He Y. (2017). Oxidized, Regenerated Cellulose Adhesion Barrier Plus Intrauterine Device Prevents Recurrence after Adhesiolysis for Moderate to Severe Intrauterine Adhesions. J. minimally invasive Gynecol. 24 (1), 80–88. 10.1016/j.jmig.2016.09.021 27742483

[B8] Cai H. Wu B. Li Y. Liu Y. Shi L. Gong L. (2019). Local Delivery of Silk-Cellulose Incorporated with Stromal Cell-Derived Factor-1α Functionally Improves the Uterus Repair. Tissue Eng. A 25 (21-22), 1514–1526. 10.1089/ten.TEA.2018.0283 30838933

[B9] Can S. Kirpinar G. Dural O. Karamustafaoglu B. B. Tas I. S. Yasa C. (2018). Efficacy of a New Crosslinked Hyaluronan Gel in the Prevention of Intrauterine Adhesions. JSLS 22 (4), e2018.00036. 10.4293/jsls.2018.00036 PMC6261745 30524185

[B10] Cao Y. Sun H. Zhu H. Zhu X. Tang X. Yan G. (2018). Allogeneic Cell Therapy Using Umbilical Cord MSCs on Collagen Scaffolds for Patients with Recurrent Uterine Adhesion: a Phase I Clinical Trial. Stem Cel Res Ther 9 (1), 192. 10.1186/s13287-018-0904-3 PMC6042450 29996892

[B11] Carbonnel M. Pirtea P. de Ziegler D. Ayoubi J. M. (2021). Uterine Factors in Recurrent Pregnancy Losses. Fertil. Sterility 115 (3), 538–545. 10.1016/j.fertnstert.2020.12.003 33712099

[B12] Carruthers A. Carruthers J. (2007). Non???Animal-Based Hyaluronic Acid Fillers: Scientific and Technical Considerations. Plast. Reconstr. Surg. 120 (6 Suppl. l), 33s–40s. 10.1097/01.prs.0000248808.75700.5f 18090341

[B13] Cervelló I. Santamaría X. Miyazaki K. Maruyama T. Simón C. (2015). Cell Therapy and Tissue Engineering from and toward the Uterus. Semin. Reprod. Med. 33 (5), 366–372. 10.1055/s-0035-1559581 26285168

[B14] Chang J. He J. Mao M. Zhou W. Lei Q. Li X. (2018). Advanced Material Strategies for Next-Generation Additive Manufacturing. Materials 11 (1), 166. 10.3390/ma11010166 PMC5793664 29361754

[B15] Chen X. Sun J. Li X. Mao L. Cui L. Bai W. (2019). Transplantation of Oral Mucosal Epithelial Cells Seeded on Decellularized and Lyophilized Amniotic Membrane for the Regeneration of Injured Endometrium. Stem Cel Res Ther 10 (1), 107. 10.1186/s13287-019-1179-z PMC6429789 30898158

[B16] Chi Y. He P. Lei L. Lan Y. Hu J. Meng Y. (2018). Transdermal Estrogen Gel and Oral Aspirin Combination Therapy Improves Fertility Prognosis via the Promotion of Endometrial Receptivity in Moderate to Severe Intrauterine Adhesion. Mol. Med. Rep. 17 (5), 6337–6344. 10.3892/mmr.2018.8685 29512784 PMC5928622

[B17] Ci L. Huang Z. Liu Y. Liu Z. Wei G. Lu W. (2017). Amino-functionalized Poloxamer 407 with Both Mucoadhesive and Thermosensitive Properties: Preparation, Characterization and Application in a Vaginal Drug Delivery System. Acta pharmaceutica Sinica B 7 (5), 593–602. 10.1016/j.apsb.2017.03.002 28924553 PMC5595263

[B18] Domiński A. Konieczny T. Kurcok P. (2019). α-Cyclodextrin-Based Polypseudorotaxane Hydrogels. Materials 13 (1), 133. 10.3390/ma13010133 PMC6982288 31905603

[B19] Du X. Hou Y. Wu L. Li S. Yu A. Kong D. (2020). An Anti-infective Hydrogel Adhesive with Non-swelling and Robust Mechanical Properties for Sutureless Wound Closure. J. Mater. Chem. B 8 (26), 5682–5693. 10.1039/d0tb00640h 32500887

[B20] Fei Z. Bin Z. Xin X. Fei H. Yuechong C. (2019). Meta-analysis on the Use of Hyaluronic Acid Gel to Prevent Recurrence of Intrauterine Adhesion after Hysteroscopic Adhesiolysis. Taiwanese J. Obstet. Gynecol. 58 (6), 731–736. 10.1016/j.tjog.2019.09.002 31759520

[B21] Fei Z. Xin X. Fei H. Yuechong C. (2020). Meta-analysis of the Use of Hyaluronic Acid Gel to Prevent Intrauterine Adhesions after Miscarriage. Eur. J. Obstet. Gynecol. Reprod. Biol. 244, 1–4. 10.1016/j.ejogrb.2019.10.018 31731019

[B22] Grover G. N. Lam J. Nguyen T. H. Segura T. Maynard H. D. (2012). Biocompatible Hydrogels by Oxime Click Chemistry. Biomacromolecules 13 (10), 3013–3017. 10.1021/bm301346e 22970829 PMC3474544

[B23] Han X. Ma Y. Lu X. Li W. Xia E. Li T.-C. (2020). Transplantation of Human Adipose Stem Cells Using Acellular Human Amniotic Membrane Improves Angiogenesis in Injured Endometrial Tissue in a Rat Intrauterine Adhesion Model. Cel Transpl. 29, 096368972095205. 10.1177/0963689720952055 PMC7784510 32838542

[B24] Han Y. Liu S. Mao H. Tian L. Ning W. (2016). Synthesis of Novel Temperature- and pH-Sensitive ABA Triblock Copolymers P(DEAEMA-co-MEO2MA-co-OEGMA)-b-PEG-b-P(DEAEMA-co-MEO2MA-co-OEGMA): Micellization, Sol-Gel Transitions, and Sustained BSA Release. Polymers 8 (11), 367. 10.3390/polym8110367 PMC6431942 30974672

[B25] Huang C. Ding D.-C. (2019). Outcomes of Adhesion Barriers in Gynecologic Surgeries. Medicine (Baltimore) 98 (50), e18391. 10.1097/md.0000000000018391 31852155 PMC6922485

[B26] Huang H. Zou L. Zhang A. Zhao X. Xu D. Xue M. (2020). A Preliminary Study on a Patented Intrauterine Stent in the Treatment of Recurrent Intrauterine Adhesions with Poor Prognosis. Ann. Transl Med. 8 (4), 57. 10.21037/atm.2020.01.77 32175351 PMC7049051

[B27] Huang X.-W. Lin M.-M. Zhao H.-Q. Powell M. Wang Y.-Q. Zheng R.-R. (2020). A Prospective Randomized Controlled Trial Comparing Two Different Treatments of Intrauterine Adhesions. Reprod. BioMedicine Online 40 (6), 835–841. 10.1016/j.rbmo.2020.02.013 32376313

[B28] Ji W. Hou B. Lin W. Wang L. Zheng W. Li W. (2020). 3D Bioprinting a Human iPSC-Derived MSC-Loaded Scaffold for Repair of the Uterine Endometrium. Acta Biomater. 116, 268–284. 10.1016/j.actbio.2020.09.012 32911103

[B29] Jian W.-H. Wang H.-C. Kuan C.-H. Chen M.-H. Wu H.-C. Sun J.-S. (2018). Glycosaminoglycan-based Hybrid Hydrogel Encapsulated with Polyelectrolyte Complex Nanoparticles for Endogenous Stem Cell Regulation in central Nervous System Regeneration. Biomaterials 174, 17–30. 10.1016/j.biomaterials.2018.05.009 29763775

[B30] Jiang C. Guo J. Cheng H. Feng Y.-H. (2019). Induced Expression of Endogenous CXCR4 in iPSCs by Targeted CpG Demethylation Enhances Cell Migration toward the Ligand CXCL12. Inflammation 42 (1), 20–34. 10.1007/s10753-018-0869-5 30105642

[B31] Kapoor S. Kundu S. C. (2016). Silk Protein-Based Hydrogels: Promising Advanced Materials for Biomedical Applications. Acta Biomater. 31, 17–32. 10.1016/j.actbio.2015.11.034 26602821

[B32] Kasiński A. Zielińska-Pisklak M. Oledzka E. Sobczak M. (2020). Smart Hydrogels - Synthetic Stimuli-Responsive Antitumor Drug Release Systems. Int. J. Nanomedicine 15, 4541–4572. 10.2147/ijn.S248987 32617004 PMC7326401

[B33] Keskin D. Mergel O. van der Mei H. C. Busscher H. J. van Rijn P. (2019). Inhibiting Bacterial Adhesion by Mechanically Modulated Microgel Coatings. Biomacromolecules 20 (1), 243–253. 10.1021/acs.biomac.8b01378 30512925 PMC6335679

[B34] Kim Y. Y. Park K.-H. Kim Y. J. Kim M. S. Liu H. C. Rosenwaks Z. (2019). Synergistic Regenerative Effects of Functionalized Endometrial Stromal Cells with Hyaluronic Acid Hydrogel in a Murine Model of Uterine Damage. Acta Biomater. 89, 139–151. 10.1016/j.actbio.2019.03.032 30898731

[B35] Kowalski G. Kijowska K. Witczak M. Kuterasiński Ł. Łukasiewicz M. (2019). Synthesis and Effect of Structure on Swelling Properties of Hydrogels Based on High Methylated Pectin and Acrylic Polymers. Polymers 11 (1), 114. 10.3390/polym11010114 PMC6401908 30960098

[B36] Kwon S. G. Kwon Y. W. Lee T. W. Park G. T. Kim J. H. (2018). Recent Advances in Stem Cell Therapeutics and Tissue Engineering Strategies. Biomater. Res. 22, 36. 10.1186/s40824-018-0148-4 30598836 PMC6299977

[B37] Lam J. Kim K. Lu S. Tabata Y. Scott D. W. Mikos A. G. (2014). A Factorial Analysis of the Combined Effects of Hydrogel Fabrication Parameters on the *In Vitro* Swelling and Degradation of Oligo(poly(ethylene Glycol) Fumarate) Hydrogels. J. Biomed. Mater. Res. 102 (10), 3477–3487. 10.1002/jbm.a.35015 PMC4012001 24243766

[B38] Larrañeta E. Stewart S. Ervine M. Al-Kasasbeh R. Donnelly R. (2018). Hydrogels for Hydrophobic Drug Delivery. Classification, Synthesis and Applications. J. Funct. Biomater. 9 (1), 13. 10.3390/jfb9010013 PMC5872099 29364833

[B39] Lee D.-Y. Lee S. R. Kim S. K. Joo J. K. Lee W. S. Shin J.-H. (2020). A New Thermo-Responsive Hyaluronic Acid Sol-Gel to Prevent Intrauterine Adhesions after Hysteroscopic Surgery: A Randomized, Non-inferiority Trial. Yonsei Med. J. 61 (10), 868–874. 10.3349/ymj.2020.61.10.868 32975061 PMC7515784

[B40] Li J. Huang B. Dong L. Zhong Y. Huang Z. (2021). WJ-MSCs Intervention May Relieve Intrauterine Adhesions in Female Rats via TGF-β1-mediated Rho/ROCK Signaling Inhibition. Mol. Med. Rep. 23 (1), 1. 10.3892/mmr.2020.11646 PMC7673328 33179074

[B41] Li X. Wu L. Zhou Y. Fan X. Huang J. Wu J. (2019). New Crosslinked Hyaluronan Gel for the Prevention of Intrauterine Adhesions after Dilation and Curettage in Patients with Delayed Miscarriage: A Prospective, Multicenter, Randomized, Controlled Trial. J. Minimally Invasive Gynecol. 26 (1), 94–99. 10.1016/j.jmig.2018.03.032 29678756

[B42] Liao Z. Liu C. Wang L. Sui C. Zhang H. (2021). Therapeutic Role of Mesenchymal Stem Cell-Derived Extracellular Vesicles in Female Reproductive Diseases. Front. Endocrinol. 12, 665645. 10.3389/fendo.2021.665645 PMC8261239 34248842

[B43] Lima C. S. A. D. Balogh T. S. Varca J. P. R. O. Varca G. H. C. Lugão A. B. Camacho-Cruz L. A. (2020). An Updated Review of Macro, Micro, and Nanostructured Hydrogels for Biomedical and Pharmaceutical Applications. Pharmaceutics 12 (10), 970. 10.3390/pharmaceutics12100970 PMC7602430 33076231

[B44] Lin J. Wang Z. Huang J. Tang S. Saiding Q. Zhu Q. (2021). Microenvironment‐Protected Exosome‐Hydrogel for Facilitating Endometrial Regeneration, Fertility Restoration, and Live Birth of Offspring. Small 17 (11), 2007235. 10.1002/smll.202007235 33590681

[B45] Lin X. Wei M. Li T. C. Huang Q. Huang D. Zhou F. (2013). A Comparison of Intrauterine Balloon, Intrauterine Contraceptive Device and Hyaluronic Acid Gel in the Prevention of Adhesion Reformation Following Hysteroscopic Surgery for Asherman Syndrome: a Cohort Study. Eur. J. Obstet. Gynecol. Reprod. Biol. 170 (2), 512–516. 10.1016/j.ejogrb.2013.07.018 23932377

[B46] Liu F. Hu S. Yang H. Li Z. Huang K. Su T. (2019). Hyaluronic Acid Hydrogel Integrated with Mesenchymal Stem Cell‐Secretome to Treat Endometrial Injury in a Rat Model of Asherman's Syndrome. Adv. Healthc. Mater. 8 (14), 1900411. 10.1002/adhm.201900411 PMC7045702 31148407

[B47] Liu H. Wang C. Li C. Qin Y. Wang Z. Yang F. (2018). A Functional Chitosan-Based Hydrogel as a Wound Dressing and Drug Delivery System in the Treatment of Wound Healing. RSC Adv. 8 (14), 7533–7549. 10.1039/c7ra13510f PMC9078458 35539132

[B48] Liu T. Weng W. Zhang Y. Sun X. Yang H. (2020). Applications of Gelatin Methacryloyl (GelMA) Hydrogels in Microfluidic Technique-Assisted Tissue Engineering. Molecules 25 (22), 5305. 10.3390/molecules25225305 PMC7698322 33202954

[B49] Liu Y. Cai J. Luo X. Wen H. Luo Y. (2020). Collagen Scaffold with Human Umbilical Cord Mesenchymal Stem Cells Remarkably Improves Intrauterine Adhesions in a Rat Model. Gynecol. Obstet. Invest. 85 (3), 267–276. 10.1159/000505691 32289792

[B50] Liu Y. Yang F. Feng L. Yang L. Chen L. Wei G. (2017). *In Vivo* retention of Poloxamer-Based *In Situ* Hydrogels for Vaginal Application in Mouse and Rat Models. Acta pharmaceutica Sinica B 7 (4), 502–509. 10.1016/j.apsb.2017.03.003 28752037 PMC5518644

[B51] López-Martínez S. Rodríguez-Eguren A. de Miguel-Gómez L. Francés-Herrero E. Faus A. Díaz A. (2021). Bioengineered Endometrial Hydrogels with Growth Factors Promote Tissue Regeneration and Restore Fertility in Murine Models. Acta Biomater. 10.1016/j.actbio.2021.08.025 34428563

[B52] Lu K.-Y. Lin Y.-C. Lu H.-T. Ho Y.-C. Weng S.-C. Tsai M.-L. (2019). A Novel Injectable *In Situ* Forming Gel Based on Carboxymethyl Hexanoyl Chitosan/hyaluronic Acid Polymer Blending for Sustained Release of Berberine. Carbohydr. Polym. 206, 664–673. 10.1016/j.carbpol.2018.11.050 30553371

[B53] Lv H. Wu B. Song J. Wu W. Cai W. Xu J. (2021). Hydrogel, a Novel Therapeutic and Delivery Strategy, in the Treatment of Intrauterine Adhesions. J. Mater. Chem. B 9, 6536–6552. 10.1039/d1tb01005k 34324619

[B54] Madl C. M. Heilshorn S. C. Blau H. M. (2018). Bioengineering Strategies to Accelerate Stem Cell Therapeutics. Nature 557 (7705), 335–342. 10.1038/s41586-018-0089-z 29769665 PMC6773426

[B55] Mancini V. Pensabene V. (2019). Organs-On-Chip Models of the Female Reproductive System. Bioengineering 6 (4), 103. 10.3390/bioengineering6040103 PMC6956296 31703369

[B56] Manna S. Ghosh A. K. Mandal S. M. (2019). Curd-Peptide Based Novel Hydrogel Inhibits Biofilm Formation, Quorum Sensing, Swimming Mortility of Multi-Antibiotic Resistant Clinical Isolates and Accelerates Wound Healing Activity. Front. Microbiol. 10, 951. 10.3389/fmicb.2019.00951 31139155 PMC6527846

[B57] Milcovich G. Lettieri S. Antunes F. E. Medronho B. Fonseca A. C. Coelho J. F. J. (2017). Recent Advances in Smart Biotechnology: Hydrogels and Nanocarriers for Tailored Bioactive Molecules Depot. Adv. Colloid Interf. Sci. 249, 163–180. 10.1016/j.cis.2017.05.009 28527520

[B58] Owusu-Akyaw A. Krishnamoorthy K. Goldsmith L. T. Morelli S. S. (2019). The Role of Mesenchymal-Epithelial Transition in Endometrial Function. Hum. Reprod. Update 25 (1), 114–133. 10.1093/humupd/dmy035 30407544

[B59] Pabuçcu E. G. Kovanci E. Şahin Ö. Arslanoğlu E. Yıldız Y. Pabuçcu R. (2019). New Crosslinked Hyaluronan Gel, Intrauterine Device, or Both for the Prevention of Intrauterine Adhesions. JSLS 23 (1), e2018.00108. 10.4293/jsls.2018.00108 PMC6400248 30846896

[B60] Pan Y. Zhao Y. Kuang R. Liu H. Sun D. Mao T. (2020). Injectable Hydrogel-Loaded Nano-Hydroxyapatite that Improves Bone Regeneration and Alveolar ridge Promotion. Mater. Sci. Eng. C 116, 111158. 10.1016/j.msec.2020.111158 32806272

[B61] Rastogi P. Kandasubramanian B. (2019). Review of Alginate-Based Hydrogel Bioprinting for Application in Tissue Engineering. Biofabrication 11 (4), 042001. 10.1088/1758-5090/ab331e 31315105

[B62] Shamloo A. Sarmadi M. Aghababaie Z. Vossoughi M. (2018). Accelerated Full-Thickness Wound Healing via Sustained bFGF Delivery Based on a PVA/chitosan/gelatin Hydrogel Incorporating PCL Microspheres. Int. J. pharmaceutics 537 (1-2), 278–289. 10.1016/j.ijpharm.2017.12.045 29288809

[B63] Sheth S. Barnard E. Hyatt B. Rathinam M. Zustiak S. P. (2019). Predicting Drug Release from Degradable Hydrogels Using Fluorescence Correlation Spectroscopy and Mathematical Modeling. Front. Bioeng. Biotechnol. 7, 410. 10.3389/fbioe.2019.00410 31956651 PMC6951421

[B64] Sun J. Mou C. Shi Q. Chen B. Hou X. Zhang W. (2018). Controlled Release of Collagen-Binding SDF-1α from the Collagen Scaffold Promoted Tendon Regeneration in a Rat Achilles Tendon Defect Model. Biomaterials 162, 22–33. 10.1016/j.biomaterials.2018.02.008 29428676

[B65] Sun X. Xue M. Deng X. Lin Y. Tan Y. Wei X. (2018). Clinical Characteristic and Intraoperative Findings of Uterine Perforation Patients in Using of Intrauterine Devices (IUDs). Gynecol. Surg. 15 (1), 3. 10.1186/s10397-017-1032-2 29386988 PMC5770510

[B66] T M. M D. a. Berrios I M. Zhu C. Gaughan C. Weinberg J. (2016). Control Release Anesthetics to Enable an Integrated Anesthetic-Mesenchymal Stromal Cell Therapeutic. Int. J. Anesth. Pain Med. 2 (1), 3. 10.21767/2471-982x.100012 PMC6519947 31106286

[B67] Tafti S. Z. G. Javaheri A. Firoozabadi R. D. Ashkezar S. K. Abarghouei H. F. (2021). Role of Hyaluronic Acid Intrauterine Injection in the Prevention of Asherman's Syndrome in Women Undergoing Uterine Septum Resection: An RCT. Int. J. Reprod. Biomed. 19 (4), 339–346. 10.18502/ijrm.v19i4.9060 33997593 PMC8106814

[B68] Tang J. Chen J. Guo J. Wei Q. Fan H. (2018). Construction and Evaluation of Fibrillar Composite Hydrogel of Collagen/konjac Glucomannan for Potential Biomedical Applications. Regen. Biomater. 5 (4), 239–250. 10.1093/rb/rby018 30094063 PMC6077832

[B69] Ugboaja J. O. Oguejiofor C. B. Igwegbe A. O. (2017). Clinico-hysteroscopic Analysis of Severe Intrauterine Adhesions Among Nigerian Infertile Women. Pan Afr. Med. J. 28, 226. 10.11604/pamj.2017.28.226.13838 29629012 PMC5881565

[B70] Wang L. Yu C. Chang T. Zhang M. Song S. Xiong C. (2020). *In Situ* repair Abilities of Human Umbilical Cord-Derived Mesenchymal Stem Cells and Autocrosslinked Hyaluronic Acid Gel Complex in Rhesus Monkeys with Intrauterine Adhesion. Sci. Adv. 6 (21), eaba6357. 10.1126/sciadv.aba6357 32494750 PMC7244313

[B71] Wang Z. Wu J. Shi X. Song F. Gao W. Liu S. (2020). Stereocomplexation of Poly(Lactic Acid) and Chemical Crosslinking of Ethylene Glycol Dimethacrylate (EGDMA) Double-Crosslinked Temperature/pH Dual Responsive Hydrogels. Polymers 12 (10), 2204. 10.3390/polym12102204 PMC7599924 32992974

[B72] Wei C. Pan Y. Zhang Y. Dai Y. Jiang L. Shi L. (2020). Overactivated Sonic Hedgehog Signaling Aggravates Intrauterine Adhesion via Inhibiting Autophagy in Endometrial Stromal Cells. Cell Death Dis 11 (9), 755. 10.1038/s41419-020-02956-2 32934215 PMC7492405

[B73] Wen H. Xiao W. Biswas S. Cong Z.-Q. Liu X.-M. Lam K. S. (2019). Alginate Hydrogel Modified with a Ligand Interacting with α3β1 Integrin Receptor Promotes the Differentiation of 3D Neural Spheroids toward Oligodendrocytes *In Vitro* . ACS Appl. Mater. Inter. 11 (6), 5821–5833. 10.1021/acsami.8b19438 30645095

[B74] Wenbo Q. Lijian X. Shuangdan Z. Jiahua Z. Yanpeng T. Xuejun Q. (2020). Controlled Releasing of SDF-1α in Chitosan-Heparin Hydrogel for Endometrium Injury Healing in Rat Model. Int. J. Biol. Macromolecules 143, 163–172. 10.1016/j.ijbiomac.2019.11.184 31765745

[B75] Wu Y. Xiang Y. Fang J. Li X. Lin Z. Dai G. (2019). The Influence of the Stiffness of GelMA Substrate on the Outgrowth of PC12 Cells. Biosci. Rep. 39 (1). BSR20181748. 10.1042/bsr20181748 30606743 PMC6340955

[B76] Xiao S. Wan Y. Zou F. Ye M. Deng H. Ma J. (2015). Prevention of Intrauterine Adhesion with Auto-Crosslinked Hyaluronic Acid Gel: a Prospective, Randomized, Controlled Clinical Study. Zhonghua Fu Chan Ke Za Zhi 50 (1), 32–36. 10.3760/cma.j.issn.0529-567x.2015.01.008 25877422

[B77] Xin L. Lin X. Zhou F. Li C. Wang X. Yu H. (2020). A Scaffold Laden with Mesenchymal Stem Cell-Derived Exosomes for Promoting Endometrium Regeneration and Fertility Restoration through Macrophage Immunomodulation. Acta Biomater. 113, 252–266. 10.1016/j.actbio.2020.06.029 32574858

[B78] Xiong Q. Zhang T. Su S. (2020). A Network Meta‐Analysis of Efficacy of Different Interventions in the Prevention of Postoperative Intrauterine Adhesions. Clin. Transl Sci. 13 (2), 372–380. 10.1111/cts.12721 31692267 PMC7070815

[B79] Xu F. Shen X. Sun C. Xu X. Wang W. Zheng J. (2020). The Effect of Mitomycin C on Reducing Endometrial Fibrosis for Intrauterine Adhesion. Med. Sci. Monit. 26, e920670. 10.12659/msm.920670 31929497 PMC6977616

[B80] Xu H.-L. Xu J. Zhang S.-S. Zhu Q.-Y. Jin B.-H. ZhuGe D.-L. (2017). Temperature-sensitive Heparin-Modified Poloxamer Hydrogel with Affinity to KGF Facilitate the Morphologic and Functional Recovery of the Injured Rat Uterus. Drug Deliv. 24 (1), 867–881. 10.1080/10717544.2017.1333173 28574291 PMC8241134

[B81] Yan Y. Xu D. (2018). The Effect of Adjuvant Treatment to Prevent and Treat Intrauterine Adhesions: A Network Meta-Analysis of Randomized Controlled Trials. J. minimally invasive Gynecol. 25 (4), 589–599. 10.1016/j.jmig.2017.09.006 28893657

[B82] Yang H. Wu S. Feng R. Huang J. Liu L. Liu F. (2017). Vitamin C Plus Hydrogel Facilitates Bone Marrow Stromal Cell-Mediated Endometrium Regeneration in Rats. Stem Cel Res Ther 8 (1), 267. 10.1186/s13287-017-0718-8 PMC5697119 29157289

[B83] Yao Q. Zheng Y.-W. Lan Q.-H. Wang L.-F. Huang Z.-W. Chen R. (2020). Aloe/poloxamer Hydrogel as an Injectable β-estradiol Delivery Scaffold with Multi-Therapeutic Effects to Promote Endometrial Regeneration for Intrauterine Adhesion Treatment. Eur. J. Pharm. Sci. 148, 105316. 10.1016/j.ejps.2020.105316 32201342

[B84] Young S. Evans-Hoeker E. (2014). Endometrial Receptivity and Intrauterine Adhesive Disease. Semin. Reprod. Med. 32 (5), 392–401. 10.1055/s-0034-1376358 24959821

[B85] Zagórska-Dziok M. Sobczak M. (2020). Hydrogel-Based Active Substance Release Systems for Cosmetology and Dermatology Application: A Review. Pharmaceutics 12 (5), 396. 10.3390/pharmaceutics12050396 PMC7284449 32357389

[B86] Zhang E. Song B. Shi Y. Zhu H. Han X. Du H. (2020). Fouling-resistant Zwitterionic Polymers for Complete Prevention of Postoperative Adhesion. Proc. Natl. Acad. Sci. USA 117 (50), 32046–32055. 10.1073/pnas.2012491117 33257542 PMC7749340

[B87] Zhang S.-S. Xia W.-T. Xu J. Xu H.-L. Lu C.-T. Zhao Y.-Z. (2017). Three-dimensional Structure Micelles of Heparin-Poloxamer Improve the Therapeutic Effect of 17β-Estradiol on Endometrial Regeneration for Intrauterine Adhesions in a Rat Model. Int. J. Nanomedicine 12, 5643–5657. 10.2147/ijn.S137237 28848344 PMC5557621

[B88] Zhang S. S. Xu X. X. Xiang W. W. Zhang H. H. Lin H. L. Shen L. E. (2020). Using 17β‐estradiol Heparin‐poloxamer Thermosensitive Hydrogel to Enhance the Endometrial Regeneration and Functional Recovery of Intrauterine Adhesions in a Rat Model. FASEB j. 34 (1), 446–457. 10.1096/fj.201901603RR 31914682

[B89] Zhao G. Cao Y. Zhu X. Tang X. Ding L. Sun H. (2016). Transplantation of Collagen Scaffold with Autologous Bone Marrow Mononuclear Cells Promotes Functional Endometrium Reconstruction via Downregulating ΔNp63 Expression in Asherman's Syndrome. Sci. China Life Sci. 60 (4), 404–416. 10.1007/s11427-016-0328-y 27921235

[B90] Zheng F. Xin X. He F. Liu J. Cui Y. (2020). Meta-analysis on the Use of Hyaluronic Acid Gel to Prevent Intrauterine Adhesion after Intrauterine Operations. Exp. Ther. Med. 19 (4), 2672–2678. 10.3892/etm.2020.8483 32256748 PMC7086218

[B91] Zhou Q. Shi X. Saravelos S. Huang X. Zhao Y. Huang R. (2021). Auto-Cross-Linked Hyaluronic Acid Gel for Prevention of Intrauterine Adhesions after Hysteroscopic Adhesiolysis: A Randomized Controlled Trial. J. minimally invasive Gynecol. 28, 307–313. 10.1016/j.jmig.2020.06.030 32681996

